# Tumor-derived exosomal miR-3157-3p promotes angiogenesis, vascular permeability and metastasis by targeting TIMP/KLF2 in non-small cell lung cancer

**DOI:** 10.1038/s41419-021-04037-4

**Published:** 2021-09-08

**Authors:** Zijian Ma, Ke Wei, Fengming Yang, Zizhang Guo, Chunfeng Pan, Yaozhou He, Jun Wang, Zhihua Li, Liang Chen, YiJiang Chen, Yang Xia

**Affiliations:** 1 Department of Thoracic Surgery, The First Affiliated Hospital with Nanjing Medical University, Nanjing, China; 2 Department of Medical Oncology, Key Laboratory of Cancer Prevention and Intervention, Ministry of Education, The Second Affiliated Hospital, Zhejiang University School of Medicine, Hangzhou, Zhejiang China

**Keywords:** Cancer microenvironment, Non-small-cell lung cancer

## Abstract

Metastasis is the main cause of death in patients with advanced lung cancer. The exosomes released by cancer cells create tumor microenvironment, and then accelerate tumor metastasis. Cancer-derived exosomes are considered to be the main driving force for metastasis niche formation at foreign sites, but the mechanism in Non-small cell lung carcinoma (NSCLC) is unclear. In metastatic NSCLC patients, the expression level of miR-3157-3p in circulating exosomes was significantly higher than that of non-metastatic NSCLC patients. Here, we found that miR-3157-3p can be transferred from NSCLC cells to vascular endothelial cells through exosomes. Our work indicates that exosome miR-3157-3p is involved in the formation of pre-metastatic niche formation before tumor metastasis and may be used as a blood-based biomarker for NSCLC metastasis. Exosome miR-3157-3p has regulated the expression of VEGF/MMP2/MMP9 and occludin in endothelial cells by targeting TIMP/KLF2, thereby promoted angiogenesis and increased vascular permeability. In addition, exosome miR-3157-3p promoted the metastasis of NSCLC in vivo.

## Background

The pre-metastatic niche is a pre-formed microenvironment formed by exosomes secreted by the primary tumor site before extensive metastasis [1–3]. It is characterized by angiogenesis, immunosuppression, inflammation, lymphangiogenesis, vascular permeability, organotropism, and reprogramming [4–7]. In addition, targeted therapy before metastasis niche may be a potential strategy for the treatment of cancer metastasis [8, 9]. It has been found that several pre-metastatic niche biomarkers are important for the diagnosis and prognosis prediction of cancer [10, 11].

Exosomes are extracellular vesicles (EVs) with a diameter of 30–150 nm [12–14]. As a key participant in cell-to-cell communication, exosomes convey information through their cargo levels, including proteins, lipids, DNA, messenger RNA, and microRNA [15–20]. Recently, it has been found that the pre-metastatic niche depends on tumor-derived exosomes [5]. The function of exosomes depends on the cell type from which they are derived [21, 22]. Studies have shown that tumor-derived exosomes participate in the exchange of genetic information between tumor cells and basal cells, resulting in the generation of a large number of new blood vessels, thereby promoting tumor growth and invasion [23, 24]. Various studies have confirmed that exosomes have played a key role in cancer tumorigenesis, growth, apoptosis, immune response, and chemotherapy resistance [25–29]. We have shown that the NSCLC-derived exosome miR-3157-3p can be transferred to vascular endothelial cells to target TIMP2/KLF2, by which to promote the angiogenesis and increase the permeability. In addition, we have demonstrated that exosome miR-3157-3p mediates the pre-metastatic niche before tumor cells transfer in nude mice by inducing angiogenesis, thereby promoting NSCLC metastasis.

## Result

### Exosomal miR-3157-3p is abundant in NSCLC patients

**Fig. 1:**
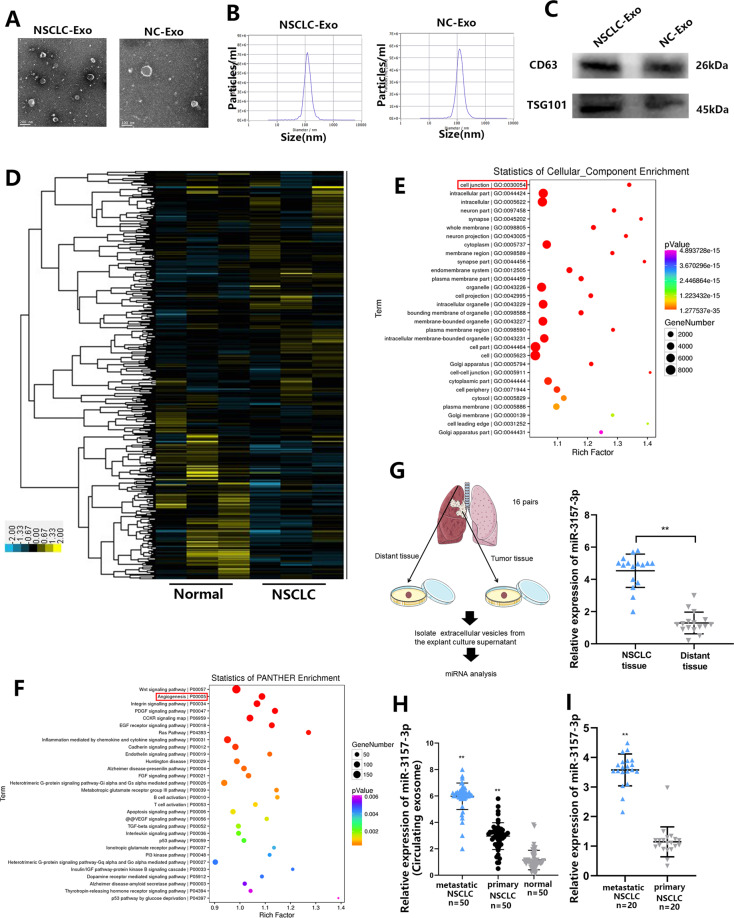
Exosomal miR-3157-3p is abundant in NSCLC patients. **A** The morphology of exosomes was examined by TEM. **B** The exosomes were quantified by nanoparticle tracking analysis. **C** CD63 and TSG101 were determined by western blot. **D** A heatmap representing microarray data for the indicated NSCLC patient plamsa exosomes with metastasis and corresponding normal plasma exosomes. Three clinical samples were pooled in each group. **E**, **F** GO enrichment analysis of DEGs: Two histograms refer to the cellular component (CC) and panther enrichment. **G** 16 pairs of explants of NSCLC tumors and distant healthy lung tissues were cultured in vitro, and the expression of miR-3157-3p of EVs isolated from the culture supernatant of explants was measured. **H** The expression levels of miR-3157-3p in plasma exosomes from NSCLC patients (50 with metastasis, 50 without metastasis and 50 noramls). **I** qRT-PCR analysis of miR-3157-3p expression in NSCLC tissues with or without metastasis (20 with metastasis and 20 without metastasis). The data are shown as the mean ± SD ( **P < 0.01).

First, exosomes were respectively extracted from plamsa of NSCLC patients (NSCLC-Exo) or healthy controls (NC-Exo). The morphology of the exosome was examined by TEM (Fig. 1A). Nanosight particle tracking shows that the size of these exosomes is about 100 nm (Fig. 1B). In addition, Western blot analysis confirmed the expression of specific exosome markers such as CD63 and TSG101 (Fig. 1C). To explore the dyregulation of exosomal miRNAs, we performed microarray (fold change > 1.5, FDR < 0.01) (Fig. 1D). Compared with normal controls, there were 44 miRNAs with abnormal expression in NSCLC plamsa exosomes, of them, 3 miRNAs (miR-3157-3p, miR-3613-5p, miR-3921) (Fig. S1A) were upregulated. The top five GO function according to *P* value were cell junction, intracellular part, intracellular, neuron part, and synapse. In the KEGG pathway analysis, the top five pathways according to *P* value was WNT signaling pathway, Angiogenesis, Integrin signaling pathway, PDGF signaling pathway and CCKR signaling pathway. GO and KEGG enrichment analysis exerted that the differential miRNAs were mainly enriched in the four categories of “Angiogenesis”, “cell junction”, “cancer” and “positive regulation biological process” (Fig. 1E, F, Fig. S1B–E). We further conducted GO-Standard analysis on the expression of differential miRNA in “biological process”, “cellar component” and “Molecular function” (Fig. S2A). Because angiogenesis and tight junctions between cells are related to the pre-metastatic microenvironment, we suspect that abnormally expressed miRNAs may be associated with metastasis. Here, we focused on investigating the role of miR-3157-3p with the highest expression. In order to exclude the possibility that the miRNA changes observed in plasma during cancer may be related to EVs derived from other sources other than tumor sources or other than tumor sources. We cultured explants of NSCLC tumors and distant healthy lung tissues in vitro, isolated EVs from the culture supernatant of explants, and detected the expression of miR-3157-3p in EVs. The tissues we use are all derived from the lobectomy of NSCLC. Because healthy tissues may be affected by tumor secretion factors, we selected healthy tissues in the distal lobes of the lung [30]. Compared with distant healthy lung tissue, the expression of miR-3157-3p in the culture supernatant EVs of the NSCLC tumor group was higher (Fig. 1G). In addition, the results of qRT-PCR showed that circulating exosomal-miR-3157-3p in NSCLC patients with metastasis was higher than without metastasis (Fig. 1H). More interestingly, we found that the miR-3157-3p in the tissues of NSCLC patients with metastasis was significantly higher than that in the tissues of patients without metastasis, and we observed that the expression of miR-3157-3p in tumor tissues and circulating exosomes was positively correlated (Fig. 1I, Fig. S2B). Collectively, the above results suggested that exo-miR-3157-3p was closely associated with metastasis. In addition, in the metastasis group, compared with the group with low plasma exo-miR-3157-3p expression, the positive rate of MMP2 and VEGF in tumor tissues in the group with high plasma exo-miR-3157-3p expression were relatively higher, while the positive rate of KLF2, TIMP2 and Occludin were lower (Fig. S2C, D).

### NSCLC transfer miR-3157-3p to HUVECs through exosomes

**Fig. 2:**
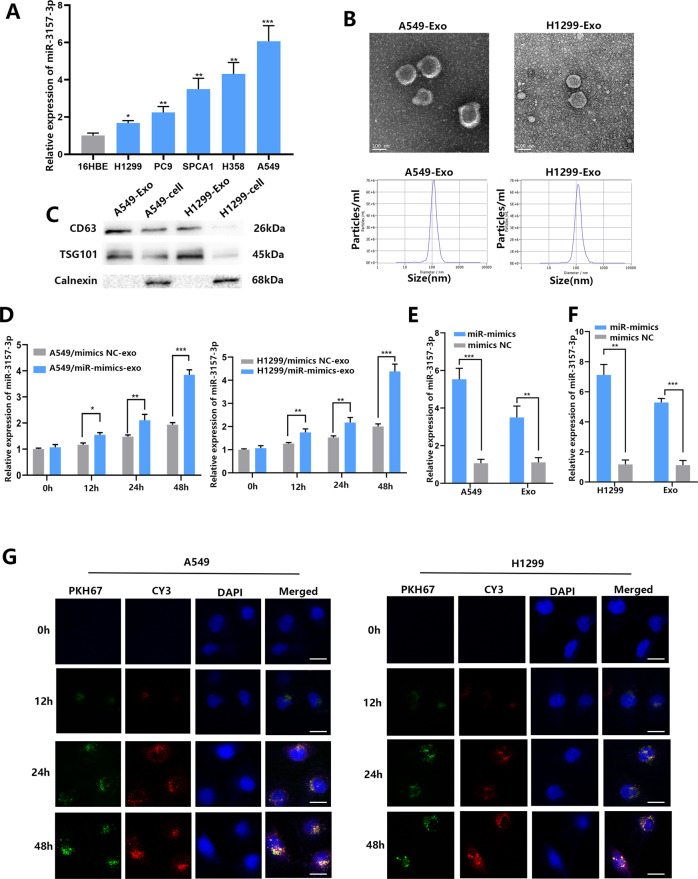
NSCLC-secreted miR-3157-3p is transferred to HUVECS. **A** NSCLC cell lines miR-3157-3p levels were analyzed using qRT-PCR. **B** Representative electron microscopy micrographs and NTA of A549 conditioning medium secreted exosomes, as well as H1299 conditioning medium secreted exosomes. Scale bar, 100 nm. **C** CD63, TSG101, and calnexin were determined by western blot. **D** qRT-PCR analysis of miR-3157-3p expression in HUVECs incubated with exosomes derived from LUAD cells for 2 h, 12 h, 24 h, and 48 h. **E**, **F** miR-3157-3p expression in A549 and H1299 transfected with miR-mimics or mimics NC) and their derived exosomes using qRT-PCR. **G** Cy3 fluorescence and PKH67 lipid dye in HUVECs after adding PKH67- labeled exosomes derived from A549 and H1299 cells for 48 h. Scale bar, 20 μm. The data are shown as the mean ± SD (*P < 0.05; **P < 0.01; ***P < 0.001).

To study the molecular mechanism of NSCLC cells regulating HUVEC, we first verified the level of miR-3157-3p in NSCLC cell lines and 16HBE cells (Fig. 2A). The exosomes were successfully isolated from the supernatants of A549 and H1299 cells, and the cup-shaped double-sided structure of 30–100 nm exosomes was displayed by transmission electron microscopy (TEM), and NTA verification was further carried out (Fig. 2B). In addition, CD63 and TSG101 are exosome markers which were used to quantify exosomes, and calnexin is not expressed in exosomes, which was determined by Western blotting. The results further confirmed that the exosomes were isolated particles (Fig. 2C). The extracted exosomes were co-cultured with HUVECs. And qRT-PCR was used to measure the expression of miR-3157-3p. qRT-PCR results showed that the expression of miR-3157-3p in HUVEC increased significantly in a time-dependent manner (Fig. 2D). A549 and H1299 transfected with lentiviral (miR-mimics) and miR-3157-3p from A549/H1299 exosomes were significantly up-regulated (Fig. 2E, F). HUVEC was incubated with PKH67-labeled exosomes derived from A549/H1299 cells, which were transfected with Cy3-labeled miR-3157-3p mimics. Cy3 fluorescence and PKH67-labeled exosomes were observed under a confocal microscope in the incubated HUVEC. The dye is distributed around the nucleus (Fig. 2G). Therefore, these findings concluded that NSCLC transferred miR-3157-3p to HUVEC through exosomes.

### Upregulation of miR-3157-3 promotes proliferation, migration, vascular leakiness and angiogenesis of HUVEC

**Fig. 3:**
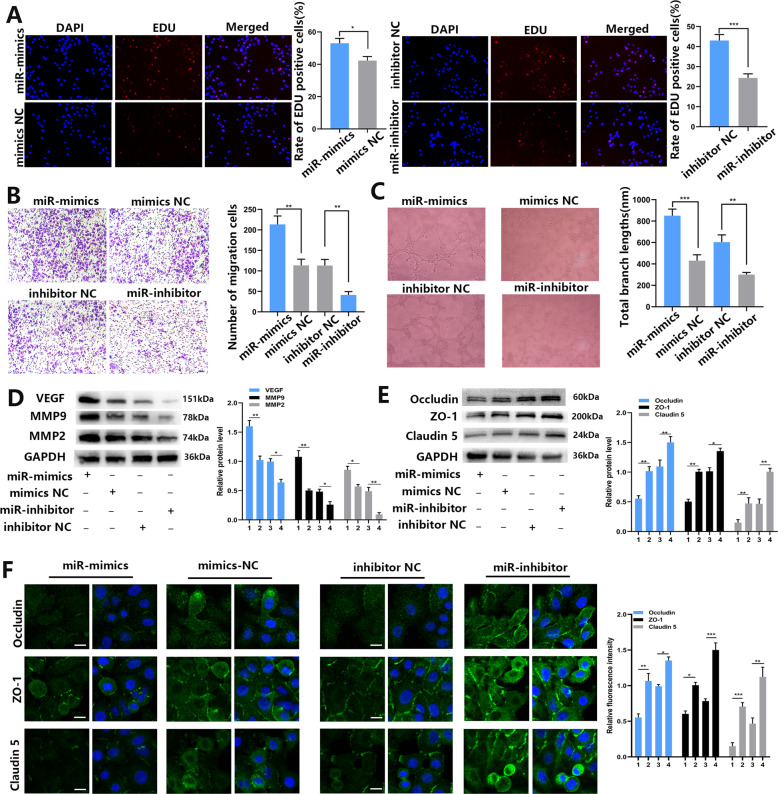
Restored miR-3157-3p enhances HUVEC proliferation, migration, tube formation, angiogenesis and permeability. **A** Proliferation of HUVECs transfected with Lv-miR-3157-3p or Lv-miR-3157-3p-inhibitor detected using EdU assay (×400); **B** migration of HUVEC transfected with Lv-miR-3157-3p or Lv-miR-3157-3p-inhibitor assessed using transwell assay (×100). **C** Tube formation of HUVECs transfected with Lv-miR-3157-3p or Lv-miR-3157-3p-inhibitor (×100). **D**, **E** Protein levels of TIMP2, VEGF, MMP2, MMP9,KLF2,ZO-1,Occludin, and Claudin5 of HUVEC transfected with Lv-miR-3157-3p or Lv-miR-3157-3p-inhibitor measured using Western blot analysis; **F** Immunofluorescence staining analysis of ZO-1, occludin, Claudin5 expression in HUVECs ransfected with Lv-miR-3157-3p or Lv-miR-3157-3p-inhibitor. Scale bar, 20 μm. 1: miR-mimics; 2: mimics NC; 3: inhibitor NC; 4: miR-inhibitor; The data are shown as the mean ± SD (*P < 0.05; **P < 0.01; ***P < 0.001).

To determine whether miR-3157-3p can affect the proliferation, migration, and angiogenesis of HUVEC in vitro, miR-3157-3p mimics or miR-3157-3p-inhibitor was transfected with HUVEC. The expression efficiency of miR-3157-3p was detected by qRT-PCR (Fig. S3A). Overexpression of miR-3157-3p enhances HUVEC proliferation, migration of vessel penetration and tube formation. In contrast, inhibiting the expression of miR-3157-3p leads to a reduction in HUVEC proliferation, migration, and tube formation (Fig. 3A–C). VEGF plays a powerful role in promoting angiogenesis. MMP2 and MMP9 can degrade type IV collagen and promote the invasion and migration of malignant tumors. ZO-1, occludin and Claudin5 are essential for the integrity of the endothelial barrier because they can enhance tight junction-related proteins. Western blot analysis showed that the upregulation of miR-3157-3p increased the protein levels of VEGF, MMP2 and MMP9, while decreased the protein levels of ZO-1, occludin and Claudin5. Inhibiting the expression of miR-3157-3p played the opposite role (Fig. 3D, E). The expression of ZO-1, occludin and Claudin5 in transfected HUVEC was further verified in the IF experiment (Fig. 3F). These findings indicated that elevated miR-3157-3p promotes HUVEC proliferation, angiogenesis and migration in vitro.

### TIMP2 and KLF2 are functional targets of miR-3157-3p in HUVECs

**Fig. 4:**
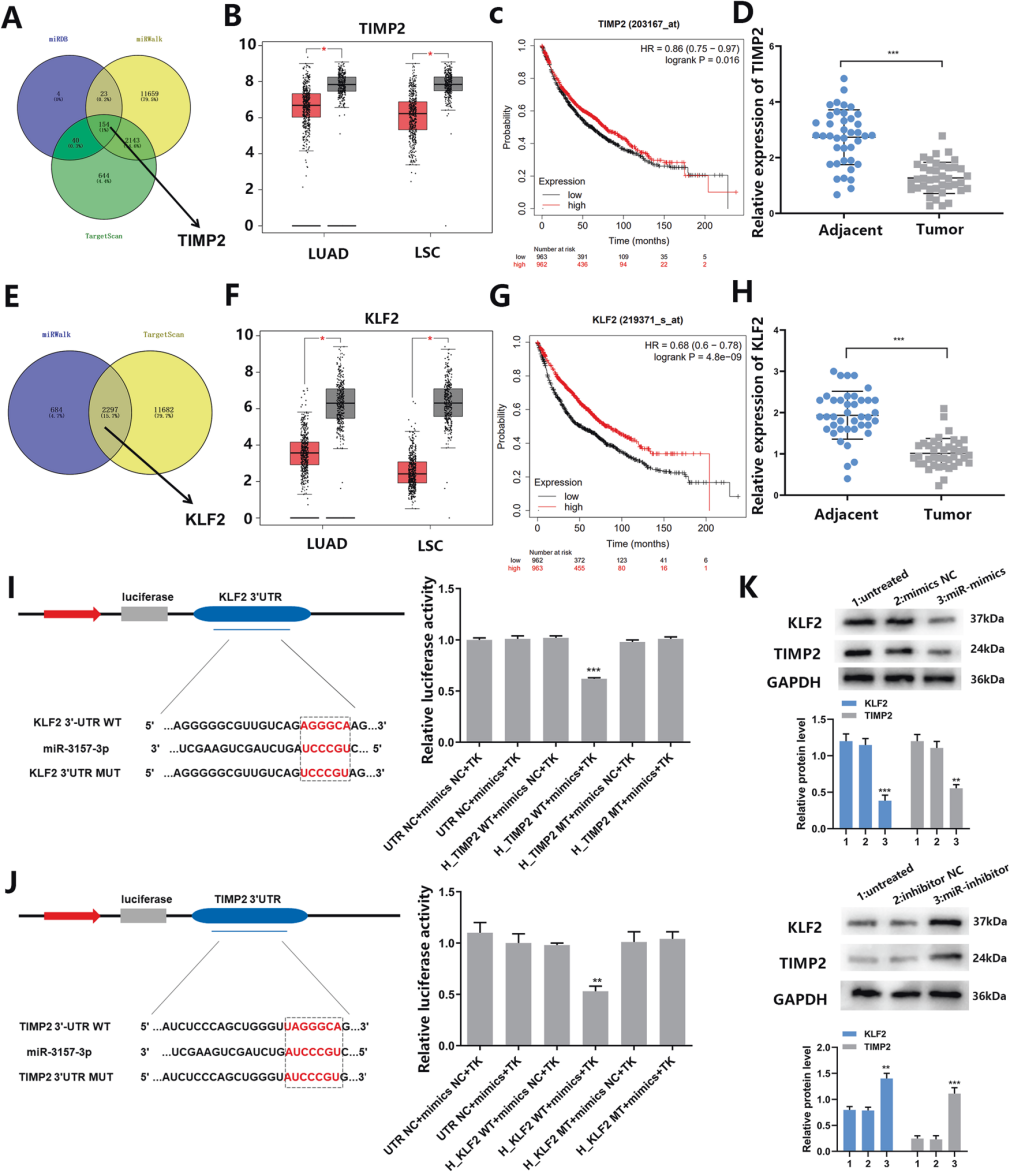
TIMP2 and KLF2 are functional targets of miR-3157-3p. **A** The candidate gene targets were predicted by intersecting outputs from four distinct prediction algorithms (TargetScan, miRDB, and miRWalk). **B** It was found that TIMP2 expression was lower in NSCLC tissues compared to normal tissues. (LUAD: lung adenocarcinoma; LSC: Lung squamous cell carcinoma; Red: tumor tissues; Gray: normal tissues). **C** The relationship between TIMP2 and the survival of NSCLC patients (*n* = 963 in the TIMP2-low group and *n* = 962 in the TIMP2-high group). **D** The TIMP2 expression level in 40 NSCLC tissues and adjacent tissues was detected by qRT-PCR. **E** The candidate gene targets were predicted by intersecting outputs from four distinct prediction algorithms (TargetScan and miRWalk). **F** It was found that TIMP2 expression was lower in NSCLC tissues compared to normal tissues. (LUAD: lung adenocarcinoma; LSC: Lung squamous cell carcinoma; Red: tumor tissues; Gray: normal tissues). **G** The relationship between KLF2 and the survival of NSCLC patients (*n* = 962 in the TIMP2-low group and *n* = 963 in the KLF2-high group). **H** The KLF2 expression level in 40 NSCLC tissues and adjacent tissues was detected by qRT-PCR. **I** The miR-3157-3p binding site in TIMP2 3′UTR and the detection of luciferase activity. **J** The miR-3157-3p binding site in KLF2 3’UTR and the detection of luciferase activity. **K** A negative regulatory effect of miR-3157-3p on TIMP2/KLF2 was tested by western blot.1: control; 2: mimics/inhibitor NC;3miR-mimics/inhibitor; The data are shown as the mean ± SD ( **P < 0.01; ***P < 0.001).

To explore how miR-3157-3p regulates angiogenesis and vascular permeability, three mRNA target prediction algorithms (miRDB, miRWalk, and Targetscan) were used to identify potential downstream targets of miR-3157-3p. Among potential targets, TIMP2 overlaps in all databases, and KLF2 overlaps in miRWalk and Targetscan (Fig. 4A, E). According to reports, TIMP2 is a natural inhibitor of MMP, inhibiting angiogenesis and invasion and metastasis. KLF2 is considered to be essential to the integrity of the endothelial barrier. Mukesh K. Jain have found that KLF2 can regulate occludin [31]. In addition, KLF2 inhibits angiogenesis by inhibiting the promoter activity of VEGF2 [32]. For the progress of the subsequent experiments, we stably transfected HUVEC cells with TIMP2/KLF2 plasmid and siRNA-TIMP2/KLF2, and verified the transfection efficiency with qRT-PCR (Fig. S3D, E). We found that overexpressed KLF2 has an inhibitory effect on angiogenesis and vascular permeability (Fig. S3F, G). Overexpressed TIMP2 has an inhibitory effect on angiogenesis, but not on vascular permeability (Fig. S3H, I). Therefore, we believe that the angiogenesis and vascular permeability produced by TIMP2 and KLF2 are critical to the metastasis of NSCLC. TIMP2 and KLF2 expression is reduced in lung cancer, and patients with decrease dexpression have a poorer outcome (Fig. 4B–D, F–H). In patients with elevated miR-3157-3p, TIMP2/KLF2 expression decreased (Fig. S3B, C). To determine whether TIMP2 and KLF2 are the targets of miR-3157-3p, we conducted a luciferase reporter gene experiment in HUVECs. As shown, TIMP2 and KLF2 3′UTR contain potential miR-3157-3p binding sites. We designed corresponding mutation sites based on their binding sites. In addition, dual luciferase reporter gene assays were performed with TIMP2/KLF2-Wt and TIMP2/KLF2-Mut co-transfected into cells with miR-3157-3p mimics or NC. Compared with the NC mimics, the luciferase activity of TIMP2-Wt and KLF2-Wt is inhibited (*p* < 0.05) in the presence of miR-3157-3p mimics, which indicates that miR-3157-3p can specifically bind TIMP2 and KLF2 (Fig. 4I, J). Western blot analysis showed that overexpressed miR-3157-3p can inhibit the expression of TIMP2 and KLF2, while inhibiting the expression of miR-3157-3p played the opposite role (Fig. 4K). In order to further verify that whether TIMP2 and KLF2 are the target genes of miR-3157-3p in HUVEC, we conducted a reversion experiment. Stably transfecting HUVEC cells with TIMP2/KLF2 plasmid and siRNA-TIMP2/KLF2 (Fig. S4A). The high expression of TIMP2 has reversed the effect of miR-3157-3p upregulation on HUVEC cell proliferation and tube formation. Similarly, the down-regulation of TIMP2 effectively counteracted the inhibitory effect of miR-3157-3p inhibitors (Fig. S4B, C). At the same time, KLF2 overexpression has the ability to inhibit the migration of miR-3157-3p to HUVECs (Fig. S4D). So we found that cells transfected with plasmid TIMP2/KLF2 showed upregulation of TIMP2/KLF2 compared with the control group, which can inhibit the effect ofmiR-3157-3p mimics. However, TIMP2/KLF2 was down-regulated in si-TIMP2/KLF2 transfected cells, inhibiting the effect of miR-3157-3p-inhibitor.

### NSCLC-secreted miR-3157-3p primes pre-metastatic niche

**Fig. 5:**
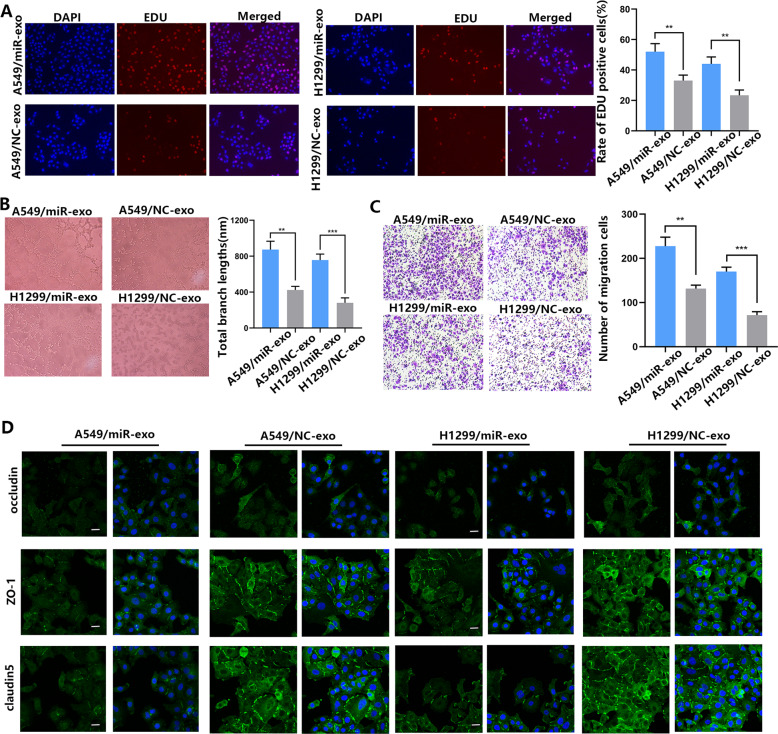
NSCLC-secreted -miR-3157-3p promotes angiogenesis and vascular leakiness in vitro. **A** Proliferation of HUVECs co-cultured with A549/miR-3157-3p exosomes, A549/NC exosomes, H1299/miR-3157-3p exosomes, H1299/NC exosomes (× 400). **B** Effects of HUVECs co-cultured with A549/miR-3157-3p exosomes, A549/NC exosomes, H1299/miR-3157-3p exosomes, H1299/NC exosomes on tube formation (x100). **C** Effects of HUVECs co-cultured with A549/miR-3157-3p exosomes, A549/NC exosomes, H1299/miR-3157-3p exosomes, H1299/NC exosomes on migration (×100). **D** Immunofluorescence staining analysis of ZO-1, occludin, Claudin5 expression in HUVECs co-cultured with A549/miR-3157-3p exosomes, A549/NC exosomes, H1299/miR-3157-3p exosomes, H1299/NC exosomes. Scale bar, 20 μm. The data are shown as the mean ± SD ( **P < 0.01; ***P < 0.001).

**Fig. 6:**
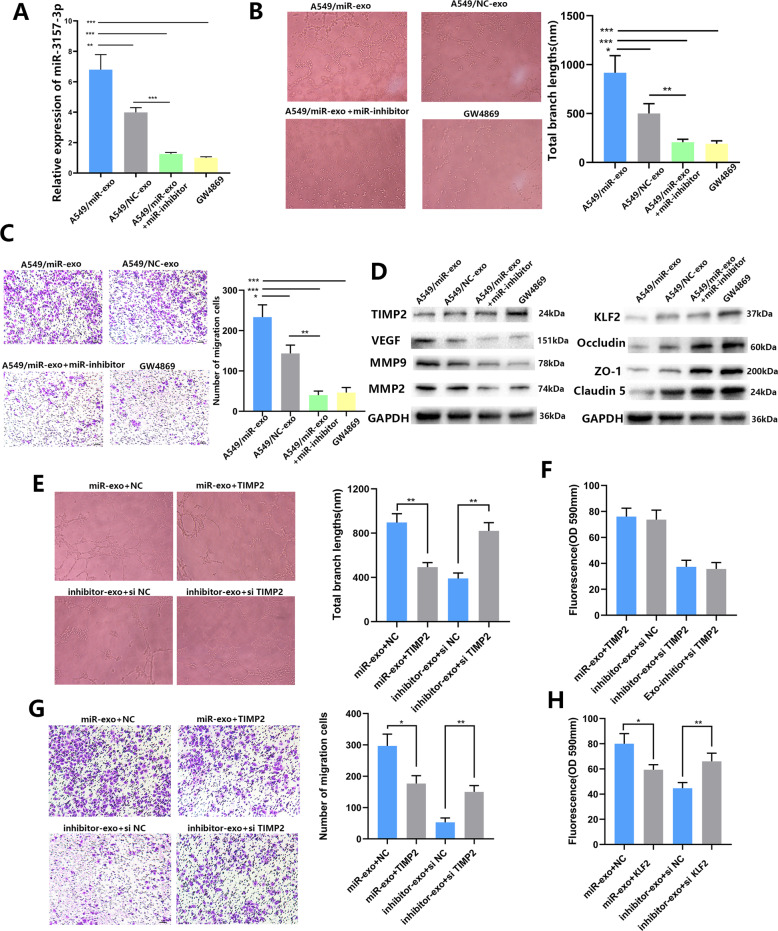
TIMP2 and KLF2 are functional targets of Exo-miR-3157-3p in HUVECs. **A** Effect of A549/miR-3157-3p exosomes, A549/NC exosomes, A549/miR-3157-3p exosomes+ miR-3157-3p inhibitor, A549/miR-3157-3p exosomes+GW4869 treatments on miR-3157-3 expression in HUVECs. **B** Effect of A549/miR-3157-3p exosomes, A549/NC exosomes, A549/miR-3157-3p exosomes+ miR-3157-3p inhibitor, A549/miR-3157-3p exosomes+GW4869 treatments on tube formation (x100). **C** Effect of A549/miR-3157-3p exosomes, A549/NC exosomes, A549/miR-3157-3p exosomes+ miR-3157-3p inhibitor, A549/miR-3157-3p exosomes+GW4869 treatments on migration (×100). **D** protein levels of TIMP2, VEGF, MMP2, MMP9,KLF2,ZO-1,Occludin, and Claudin5 of HUVEC treated with A549/ miR-3157-3p exosomes, A549/NC exosomes, A549/miR-3157-3p exosomes+miR-3157-3p inhibitor, A549/miR-3157-3p exosomes+GW4869 measured using Western blot analysis (**E**, **G**). The effect of promoting tube formation and migration of Exo- miR-3157-3p was reversed by high expression of TIMP2, while knockdown of TIMP2 enhanced the role of Exo-miR-3157-3p. **F** TIMP2 cannot reverse the role of Exo-miR-3157-3p on permeability. **H** The effect of p permeability of Exo-miR-3157-3p was reversed by high expression of KLF2, while knockdown of KLF2 enhanced the role of Exo-miR-3157-3p. The data are shown as the mean ± SD (*P < 0.05; **P < 0.01; ***P < 0.001).

We speculate that exosome miR-3157-3p can significantly promote the proliferation, migration, angiogenesis and increased permeability in HUVECs in a manner similar to endogenous miR-3157-3p, and the formation of pre-metastatic niche. Then, we conducted a series of experiments to further confirm whether exosome miR-3157-3p induces angiogenesis and vascular permeability. The exosomes isolated from transfected NSCLCs were co-cultured with HUVECs in order to determine whether exosomal miR-3157-3p mediates angiogenesis and permeability of HUVECs. Compared with the NC-Exos, miR-3157-3p-Exos can significantly increase HUVEC angiogenesis and vascular permeability. Related processes include HUVEC proliferation (Fig. 5A), tube formation (Fig. 5B), and migration (Fig. 5C). The expressions of ZO-1, Occludin, and Claudin5 were further studied in the IF experiment (Fig. 5D). To further determine that miRNAs in exosomes play a role in promoting metastasis, we used PCR to study the expression level of miR-3157-3p in each group (Fig. 6A). In addition, treatment with miR-3692-3p inhibitor and GW4869 (an inhibitor of exosome generation), A549/miR-3157-3p-Exos fail to induces angiogenesis and migration of HUVECs (Fig. 6 B, C). We have confirmed in WB and found that in HUVEC cells with high miR-3157-3p expression, TIMP2, KLF2, ZO-1, Occludin, Claudin5 expression decreased, while VEGF, MMP2, MMP9 expression increased (Fig. 6D). In order to further study whether TIMP2 and KLF2 are miR-3157-3p-Exo target genes in HUVEC, we conducted a rescue experiment. We stably transfected HUVEC cells with TIMP2/KLF2 plasmid and siRNA-TIMP2/KLF2. The high expression of TIMP2 reversed the effect of miR-3157-3p-Exo regulation on HUVEC cell tube formation and migration. Similarly, the down-regulation of TIMP2 effectively counteracted the inhibitory effect of miR-3157-3p inhibitors-Exos (Fig. 6E, G). The expression of TIMP2 has no effect on vascular permeability (Fig. 6F). In addition, the upregulation of KLF2 inhibited miR-3157-3p-Exos vascular penetration, while the low expression of KLF2 had an opposite effect (Fig. 6H).

### Exosomal miR-3157-3p promotes angiogenesis and vascular leakiness in vivo

**Fig. 7:**
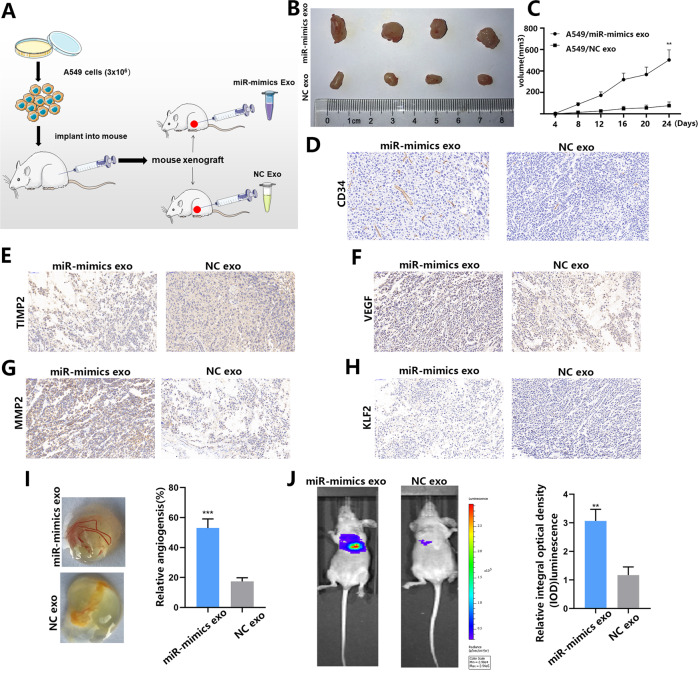
Exosomal miR-3157-3p promotes angiogenesis and vascular leakiness in vivo. **A**, **B** After 7–9 days, subcutaneous mouse xenografts were intratumourally injected with miR-3157-3p-mimics Exo or NC-Exo (*n* = 4 each). **C** Tumor volume. **D**–**H** CD34, TIMP2, VEGF, MMP2 and KLF2 expression levels the samples collected from nude mice were analyzed by IHC. **I** the number of branches of blood vessels (5 mm) around the CAM vehicle; **J** The mice were injected with A549 cells via tail vein after exposure to miR-3157-3p-mimics-Exo or NC-Exo treatments. IVIS Lumina II system was used to detect lung metastatic nodule of nude mice. The data are shown as the mean ± SD ( **P < 0.01; ***P < 0.001).

We have established a mouse xenograft model to assess angiogenesis in vivo (Fig. 7A). Consistently, miR-3157-3p-Exos-treated mouse xenografts resulted in higher microvessel density and larger tumor size compared to NC-Exos treatment (Fig. 7B–D). IHC staining found that compared to the control group, TIMP2 and KLF2 decreased in the miR-3157-3p-Exos treatment group, while the expression of VEGF and MMP2 increased (Fig. 7F–H). To further confirm that NSCLC cells affect angiogenesis in vivo, a chicken chorioallantoic membrane (CAM) model was established to study the effect of exosomes miR-3157-3p on angiogenesis in vivo. The overexpression of miR-3157-3p enhanced angiogenesis, and the number of blood vessel branches (5 mm) around the CAM vehicle increased significantly (Fig. 7I). These results then showed that exosome miR-3157-3p significantly enhanced angiogenesis in vitro and in vivo. In order to study whether the vascular permeability induced by miR-3157-3p-Exos can promote the transfer of NSCLC, exosomes isolated from transfected A549 cells were injected into the tail vein of nude mice. As expected, mice injected with A549/miR-3157-3p-Exos had more lung metastatic nodules than mice derived from A549/NC-Exos (Fig. 7J). Taken together, these results clearly show that cancer-derived exosome miR-3157-3p can induce the formation of a niche before metastasis, thereby promoting the metastasis of NSCLC.

### Exosomal miR-3157-3p is associated with metastatic progression and microvessel density in NSCLC patients

**Fig. 8:**
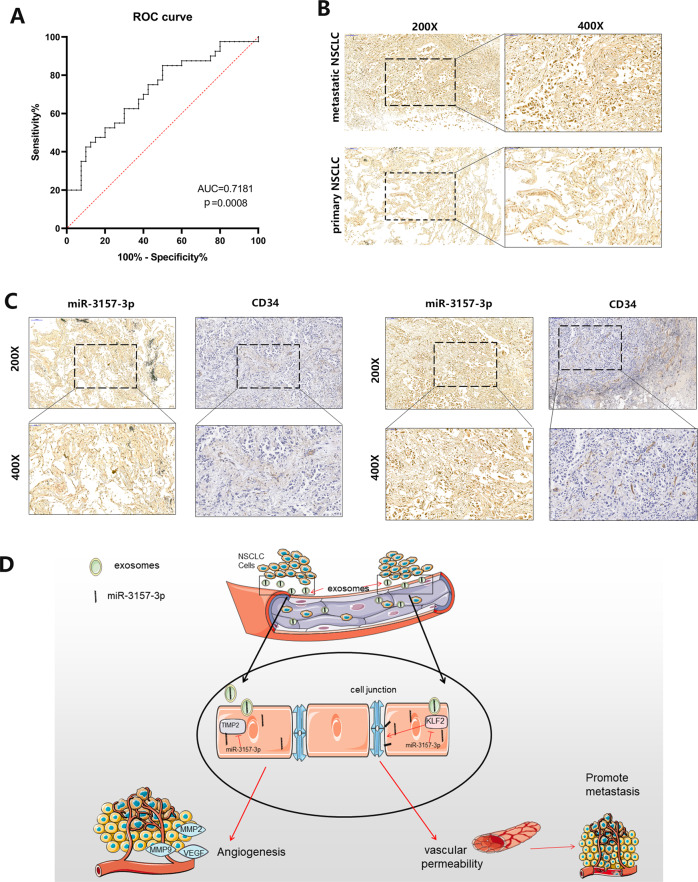
Exosomal miR-3157-3p is associated with NSCLC metastasis. **A** ROC curves were used to evaluate whether exosomal‐miR‐3157-3p acts as a biomarker. **B** Representative images of in situ hybridization for miR-3157-3p in the tissues with or without metastasis. **C** Representative images of miR-3157-3p IHC for CD34 with high or low expression levels of miR-3157-3p. The scale bar in 200× images represents 100 µm. The scale bar in 400× images represents 50 µm. **D** Schematic diagram of the role of NSCLC-secreted miR- 3157-3p in pre-metastatic niche formation. The data are shown as the mean ± SD.

In previous experiments we have confirmed that circulating exosomes are significantly elevated in the blood of metastatic patients compared to nonmetastatic patients. To assess whether exosome miR-3157-3p acts as a biomarker, we performed receiver operating characteristic curves. The area under the curve was 0.7181 in exosome miR-3157-3p (Fig. 8A). Through ISH experiments, we found that miR-3157-3p in metastatic lung cancer tissues was higher than non-metastatic lung cancer tissues (Fig. 8B). Interestingly, we found that in tissues with high miR-3157-3p expression, CD34 expression also increased. We suspect that miR-3157-3p will promote angiogenesis in cancer tissue (Fig. 8C). Therefore, our clinical data indicate that high levels of miR-3157-3p in circulating exosomes are associated with NSCLC metastasis.

## Discussion

Lung cancer is the malignant tumor that causes the most deaths among cancers, and metastasis is the main cause of death of patients [33]. The niche before metastasis is the microenvironment prepared for colonizing circulating tumor cells in specific organs, including inflammation, immune response, angiogenesis, organic matter, matrix remodeling, and biomarker expression. The microenvironment before metastasis can increase angiogenesis and vascular permeability, thereby promote the metastasis. Studies have showed that exosomes released by hypoxic tumors are more likely to cause angiogenesis and vascular leakage [33, 34]. Cancer-induced vascular permeability and angiogenesis have played a key role in cancer metastasis. Exosomes from NSCLC can be used as a communication medium between cells. MiR-3157-3p secreted from NSCLC can induce a niche before metastasis by promoting angiogenesis and destroying the tight junction of venous endothelial cells.

Exosomes are EVs with a diameter of 30–150 extranm. The contained miRNA can regulate tumor immunity and microenvironment, which may promote tumor invasion, metastasis, and angiogenesis. GSC-derived exosomes such as overexpressing miR-26a can promote HBMEC proliferation and angiogenesis in vitro by inhibiting PTEN [35]. Exosome miR-25-3p is involved in niche formation before the transfer and may be used as a blood-based biomarker for CRC transfer [36]. Therefore, exosome miRNA plays an important role in regulating cancer progression [37].

miR-3157-3p is abnormally expressed in NSCLC patients, especially those with metastases. We found that miR-3157-3p in vascular endothelial cells can induce vascular permeability and angiogenesis by down-regulating KLF2 and TIMP2. Kruppel-like factors (KLFs) are a subclass of the zinc finger family of DNA-binding transcription factors. KLF2 can regulate the key tight junction protein occludin in endothelial cells to maintain the integrity of the endothelial barrier function. TIMP2 is a natural inhibitor of MMP activity and inhibits angiogenesis. In NSCLC, both KLF2 and TIMP2 are down-regulated. Our results showed that miR-3157-3p in HUVEC can absorb exosomes downregulates KLF2 and TIMP2, and subsequently reduce its downstream target, occludin, and increase the levels of VEGF/MMP2/MMP9. Our data indicate that the exosome miR-3157-3p secreted by NSCLC promotes vascular permeability and angiogenesis by silencing KLF2 and TIMP2 (Fig. 8D).

Tumor-derived exosomes are considered to be the main driving force of the pre-metastatic niche. Exosomes participated in angiogenesis and increased vascular permeability, thereby promote the formation of niche before transfer. Before metastasis, a microenvironment suitable for tumor metastasis has been created for tumor metastasis. In this study, we explored that whether exosome miR-3157-3p can regulate niche formation before metastasis. Cancer-induced vascular permeability/angiogenesis is one of the characteristics of the pre-metastatic niche. In prostate cancer (PCa), miR-21-5p and miR-139-5p, which are microRNAs derived from exosomes, could coordinately regulate the ecological niche before metastasis, and was highly relevant to differentiate PCa carcinogenesis, fibroblast proliferation, differentiation, migration, and angiogenesis [38]. In vitro permeability measurements showed that the exosome miR-25-3p of NSCLC cells significantly promoted HUVEC permeability and angiogenesis, reduced the levels of KLF2, ZO-1, occludin, Claudin5, and increased VEGF/MMP2/MMP9 level. We also found that the upregulation of miR-3157-3p in NSCLC cells promoted proliferation, angiogenesis, and metastasis. Otherwise, we have proposed a new role of TIMP2/KLF2 in the formation of cancer-induced pre-metastatic niche, and provided new insights into the formation of the metastasis niche.

However, the limitations of this study are: (a) lack of large clinical samples; (b) In this study, we have prevented the transport of exosomes to recipient cells may be an effective strategy to prevent tumor metastasis. But there are many targets that can be used to inhibit the formation of pre-metastatic niche, for example, the prevent of the production of pro-inflammatory factors, the inhibition of the recruitment of BMDC, the prevent of angiogenesis and vascular penetration, and the destroy of local matrix and reactivating anti-tumor immune responses. These goals may develop potential methods for preventing and controlling cancer metastasis in future. (c) In vivo experiment, we preliminarily demonstrated that Exo-miR-3157-3p promotes angiogenesis and vascular leakiness via nude mice treated with A549 and corresponding exosomes. However, use of a nude mouse model devoid of any functional immune system, leaves the in vivo observations not fully representative of a real life scenario. Thus, the functions of Exo-miR-3157-3p involved in NSCLC progression require deeper and more comprehensive exploration.

In summary, we illustrated that blocking exosome miR-3157-3p from NSCLC cells can maintain the tight junction between vascular endothelium, reduce pulmonary vascular permeability and subsequent NSCLC metastasis, which indicates that miR-3157-3p can be used as a therapeutic target for the intervention in NSCLC metastasis.

## Methods

### Cell lines and human tissue samples

All plasma samples were collected from NSCLC patients (50 without metastasis and 50 with metastasis) in our hospital between April 2013 and June 2019, and age-matched healthy human plasma samples (*n* = 50) were selected from the Physical Examination Center of our hospital. The blood sample was then centrifuged at 2500 *g* for 10 m to extract the plasma and stored at −80 °C. We have collected specimens of 40 patients (20 without metastasis and 20 with metastasis) who underwent radical surgery between 2013 and 2019. Fresh biopsy tissue is frozen and stored in liquid nitrogen until being used. The explants of tumor tissues and distant normal lung tissues were cultured in vitro. After removing the visceral pleura, the tissue was cut into small pieces and cultured in serum-free RPMI for 24 h, and added with penicillin and streptomycin (100 mg/ml). Exosomes were isolated from the culture supernatant of explants, and detected the expression of miR-3157-3p. Each patient had signed an informed consent form and this was approved by the Ethics Committee of the First Affiliated Hospital of Nanjing Medical University.

This study involved four human NSCLC cell lines (H1299, SPCA1, PC9, A549), one human bronchial epithelioid cell line (16HBE) and HUVEC (human umbilical vein endothelial cells). All cell lines were purchased from Shanghai Academy of Sciences. (H1299, SPCA1, PC9, A549 were cultured in DMEM medium (GIBCO, Gaithersburg, USA) supplemented with 10% fetal bovine serum. HUVEC was cultured in F12-K medium (GIBCO with 10% fetal bovine serum, Gaithersburg, USA). 16HBE was cultured in Defined Keratinocyte SFM medium (GIBCO, Gaithersburg, USA) containing 10% fetal bovine serum (HyClone, Logan, USA).

### Microarray analysis

The miRNA 4.0 Array of Affymetrix was used to analyze the miRNA expression in plasma exosomes of healthy people (normal) and in NSCLC patients with lymph node metastasis. What we have done has obtained the approval from the First Affiliated Hospital of Nanjing Medical University (Nanjing, China). Informed consent was signed by each patient on the day of admission. Three clinical samples were collected from each group. The miRNA array experiment was conducted in the microarray core laboratory of Beijing Boao Jingdian Biotechnology Co., Ltd. (Beijing, China).

### Cell transfection

Lentiviral vectors that over-express miR-3157-3p (miR-mimics) and repress miR-3157-3p (miR-inhibitor) were constructed and generated. Mimics and inhibitor were purchased from GiKai GENE (Shanghai, China) and transfected according to the manufacturer’s instructions. Plasmid TIMP2/KLF2, empty vector, si-TIMP2/si-KLF2 were purchased from Gene Pharma (Shanghai, China). Transfection was performed using elipofectamine 3000 reagent (Invitrogen) according to the manufacturer’s instructions.

### PCR

To determine the expression level of miR-3157-3p, total RNA was isolated and extracted from tissues and cells by TRIzol reagent (Invitrogen, CA, USA) according to the instructions. CDNA was generated by reverse transcription using total RNA and PrimeScriptRT reagent (Takara, Kusatsu, Japan), and detected using SYBR Green (Takara) at ABI StepOnePlus real-time quantitative PCR instrument (StepOnePlus, ABI Company, Oyster Bay, NY, USA). Taking GAPDH and U6 as endogenous controls, mRNA and miRNA were normalized. The 2−ΔΔCT method was used to quantify the relative levels of mir-3157-3p and TIMP2/KLF2. Each sample is in triplicate. miRNA quantification: Bulge-loopTM miRNA qRT-PCR Primer Sets (one RT primer and a pair of qPCR primers for each set) specific for miR-3157-3p is designed by RiboBio (Guangzhou, China).

### Isolation and identification of exosomes

NSCLC cells were cultured in DMEM medium supplemented with 10% fetal bovine serum without exosomes. The exosomes were separated from the cell culture medium by differential centrifugation. All steps were performed at 4 °C. First, after centrifugation at 500 *g* and 10 min to remove cells and other debris, the supernatant was centrifuged at 16,000 *g* for 30 m. Finally, the supernatant was centrifuged at 110,000 *g* for 70 m. The EXOQ5A-1 EXOTC10A-1 Kit (SBI,Shanghai) was used to isolate plamsa exosomes according to the manufacturer’s instructions. And the amount of exosomes was measured by the BCA protein assay kit (KeyGEN BioTECH). For TEM, 5–10 u extracted exosomes are placed on the copper carrier grid. Add 10–20 μl of EM solvent dropwise, and carefully suck up with clean filter paper after 1 min, then observe the exosomes with Philips CM120 biological dual transmission electron microscope (FEI Company, USA). The method of Nanoparticle-tracking analysis is used to analyze the size of exosomes. The exosomes (10–20 mg) in 1 mL PBS and vortex were dissolved for 1 min to distribute the exosomes evenly. Then, the NanoSight Nanoparticle Tracking Analyzer (NTA, Malvern Analysis, UK) was used to measure and observe the scale distribution of the exosomes. For exosome labeling, PKH67 membrane dye (Sigma) was used to fluorescently label exosomes according to the instructions.

### Dual luciferase report

The 3′UTR fragments of KLF2 and KLF4 genes were amplified and inserted into the vector. Then complete co-transfection of TIMP2 and KLF2 3′UTR plasmids with miR-3157-3p lentiviral vector into cells by using Lipofectamine 2000 (Invitrogen USA). 48 h after the transfection, Dual-Lumi^™^ dual luciferase reporter gene detection kit (Biyuntian: RG088S) detects dual luciferase. The Renilla activity was taken as a normalization of luciferase activity. All measurements were performed in triplicate, and each experiment was repeated three times.

### Western blotting

The total protein was extracted from the cells with the RIPA reagent (Beyotime, Shanghai, China) who contains 10 μg/mL Phosphatase inhibitor and 100 μg/mL PMSF (Beyotime, Shanghai, China). The protein were separated by sodium dodecyl sulfate polyacrylamide gel electrophoresis (SDS-PAGE) and then transferred onto polyvinylidene difluoride membrane. After sealing for 1 h, the membrane were diluted with primary antibody diluted, TIMP2 (SAB, #41500, 1: 500 dilution) vascular endothelial growth factor (VEGF) (SAB, #320217, 1: 500 dilution) (MMP2) (Abcam, ab92536, 1: 1000 dilution), MMP-9 (Abcam, ab76003, 1: 1000 dilution), ZO-1(Abcam, ab96587, 1: 500 dilution), occludin(Abcam, ab216327, 1: 1000 dilution), claudin 5 (GeneTex, GTX00796, 1: 500 dilution) and GAPDH (Abcam, ab181602, 1: 10000 dilution) overnight at 4 °C. The TBST was washed 5 m for three times and then incubated for 2 h in the corresponding secondary antibody. After which, it was developed by enhanced chemiluminescence (ECL).

### EDU assay, migration assay, and angiogenesis assay

From EDU we know that spread the transfected cells evenly in a 24-well plate with 3 × 10^4^ cells per well, then use the EdU Apollo567 extracorporeal flow cytometry kit (Guangzhou RiboBio) to perform EdU according to the instructions, by which we can acquire images with a fluorescence microscope. From recruitment assay, we get the following result: ~2 × 10^4^ cells in serum-free medium (400 μl) were seeded into the upper Transwell chamber for migration assay, who use 8.0 μm Transwell Permeable Supports (Corning, New York, USA). The lower chamber was supplemented with 600 μl DMEM medium containing 10% FBS, and was incubated at 37 °C for 24 h. Remaining cells in the upper membrane were cleared with Cotton swabs, and they were stained with paraformaldehyde and 0.1% crystal violet (Beyotime, Shanghai, China) for 20 m at 37 °C.

Tube formation: Spread 250 u Matrigel (BD Bioscience, USA) on a 24-well plate and place in an incubator for half an hour. Approximately 1 × 10^5^ treated HUVEC cells were resuspended in each well of a 24-well plate. Tube formation was induced at 37 °C for 8 h. Then we observe the tube length with a microscope. From CAM, we get the result: Incubate 20 fertilized eggs at 37.5 °C and 70% humidity for 8 days, and an artificial airbag was created, also a small window was cut in the shell above the artificial airbag. We use exo-miR-3157-3p-mimics, exo-NC mixed with equal volume of Matrigel (BD Biosciences, Bedford, MA) to inject into CAM. The area around the implanted Matrigel was photographed, and the number of blood vessels was obtained by counting the branches of the blood vessels [39]. Each experiment was repeated three times. From the in vitro permeability measurement, we get the following result: Rhodamine B isothiocyanate-dextran (Sigma) was added to the top well of the transwell filter, and HUVEC (10^5^ cells per well) was treated on it for 3 days. Then collect the culture medium in the bottom well after 30 m and monitor the appearance of fluorescence under 544 nm excitation and 590 nm emission.

### Animal experiment

The six-week-old male nude mouse was purchased from the Animal Center of Nanjing Medical University (Nanjing, China) and what we have done was approved by the Animal Ethics Committee of Nanjing Medical University. The exosomes involved in the animal experiments were obtained from the above-mentioned ultracentrifugation, and the concentration of exosomes was diluted to about 6.5E+7 Particles/mL. Nude mice were randomly divided into four groups, four in each group. The 3 × 10^6^ A549 cell samples were injected subcutaneously into the groin area of BALB/c nude mice. After 9 days, 5 µg of exosomes were injected into xenografts every other day. Three days after the last injection (a total of 18 days), the tumor tissue was dissected, fixed in 10% formalin, and embedded in paraffin for further study. For the determination of tail vein metastasis, 6-week-old nude mice were injected with 5 µg exosomes through the tail vein every other day for 2 weeks. The 2 × 10^6^ A549 cells were injected into the tail vein of nude mice treated with exosomes. After 60 days, the mice were sacrificed, and the transfer node was checked using the IVIS Lumina II system.

### Statistical analysis

All statistical analyses were performed by spss 19.0 and GraphPad software 8.0. The *P* values were analyzed with Student’s *t* test, one-way ANOVA. We select for statistical significance when *P* is <0.05.

## Supplementary information

supplementary figure legend

Supplementary figure1

Supplementary figure2

Supplementary figure3

Supplementary figure4
