# Supplementary material for: Tumor-derived exosomal miR-3157-3p promotes angiogenesis, vascular permeability and metastasis by targeting TIMP/KLF2 in non-small cell lung cancer
Source: Cell Death Dis. 2021 Sep 8;12(9):840. doi: 10.1038/s41419-021-04037-4 (PMC8426367; doi:10.1038/s41419-021-04037-4)
Supplement: Supplementary file 1 — supplementary figure legend [file 41419_2021_4037_MOESM1_ESM.docx]

**Supplementary Figure 1.** MiR-3157-3p is a metastasis-associated miRNA in NSCLC.**A** Three miRNAs (miR-3157-3p, miR-3613-5p, miR-3921) were significantly up-regulated in Microarray analysis. **B-C** GO enrichment analysis of DEGs: Two histograms refer to the panther and cellular component (CC) enrichment. **D-E** GO and KEGG enrichment analysis of DEGs: Two histograms refer to the disease and biological process (BP) enrichment, respectively.

**Supplementary Figure 2. A** GO-standard analysis of DEGs: histograms refer to the biological process (BP), cellular component (CC) and molecular function (MF) enrichment, respectively. **B** Spearman’s correlation analysis between the miR-3157-3p levels in NSCLC tissues and the miR-3157-3p levels in circulating exosomes. *n* = 20. **C-D**. Splitting the metastatic group into high/low plasma exo-miR-3157-3p expression and examine the two groups of tissue samples by IHC for KLF2, MMP2, VEGF, TIMP2 and Occludin.

**Supplementary Figure 3:** The role of TIMP2 and KLF2 in HUVECs. **A** HUVECs were transfected with miR-3157-3p mimics, mimics NC, miR-3157-3p inhibitor or inhibitor NC, and the expression level of miR-3157-3p was detected by qRT-PCR.**B** correlation analysis of miR-3157-3p expression with KLF2 expression. **C** correlation analysis of miR-3157-3p expression with TIMP2 expression. **D-E** The expression levels of KLF2 and TIMP2 were verified in the transfected HUVECs by qRT-PCR. **F** Effect of KLF2 over-expression on tube formation ability of HUVECs by tube formation assay. **G** Effect of KLF2 over-expression on permeability of HUVEC monolayers by in vitro permeability assay. **H** Effect of TIMP2 over-expression on tube formation ability of HUVECs by tube formation assay. **I** Effect of TIMP2 over-expression on permeability of HUVEC monolayers by in vitro permeability assay. The data are shown as the mean ± SD (**P* < 0.05; ***P* < 0.01, ****P* < 0.001).

**Supplementary Figure 4:** TIMP2 and KLF2 are functional targets of miR-3157-3p in HUVECs. **A** The expression levels of TIMP2/KLF2 protein after TIMP2/KLF2 plasmid and si-TIMP2/KLF2 transfection were analysed by Western blotting in co-transfected cell lines. **B, C** The effect of promoting proliferation and tube formation of miR-3157-3p was reversed by high expression of TIMP2, while knockdown of TIMP2 enhanced the role of miR-3157-3p. **D** The effect of migration of miR-3157-3p was reversed by high expression of KLF2, while knockdown of KLF2 enhanced the role of miR-3157-3p. The data are shown as the mean ± SD (**P* < 0.05; ***P* < 0.01, ****P* < 0.001).
