# Supplementary figures and images for: Tumor-derived exosomal miR-3157-3p promotes angiogenesis, vascular permeability and metastasis by targeting TIMP/KLF2 in non-small cell lung cancer
Source: Cell Death Dis. 2021 Sep 8;12(9):840. doi: 10.1038/s41419-021-04037-4 (PMC8426367; doi:10.1038/s41419-021-04037-4)

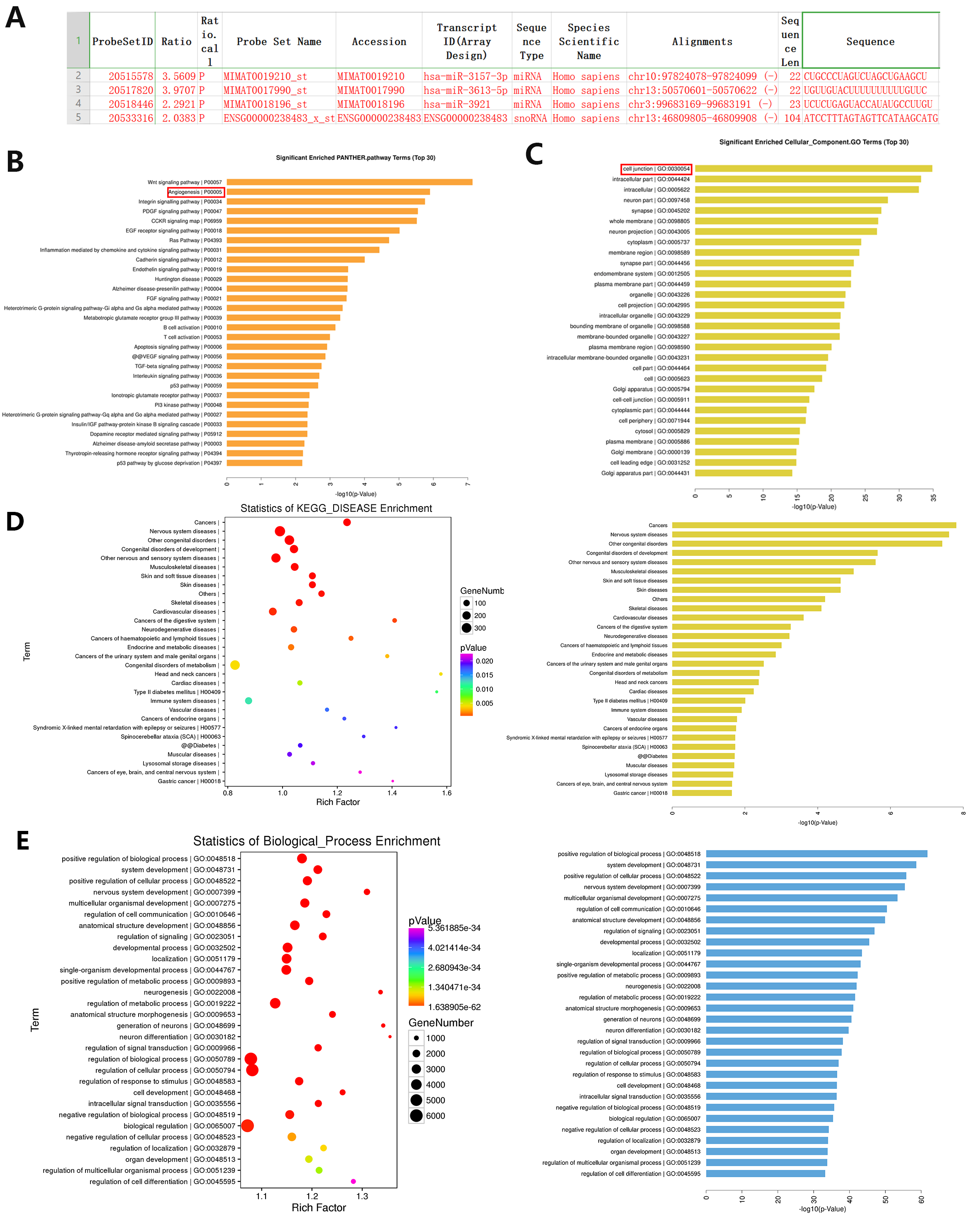

Supplement: Supplementary file 2 — Supplementary figure1 [file 41419_2021_4037_MOESM2_ESM.tif]

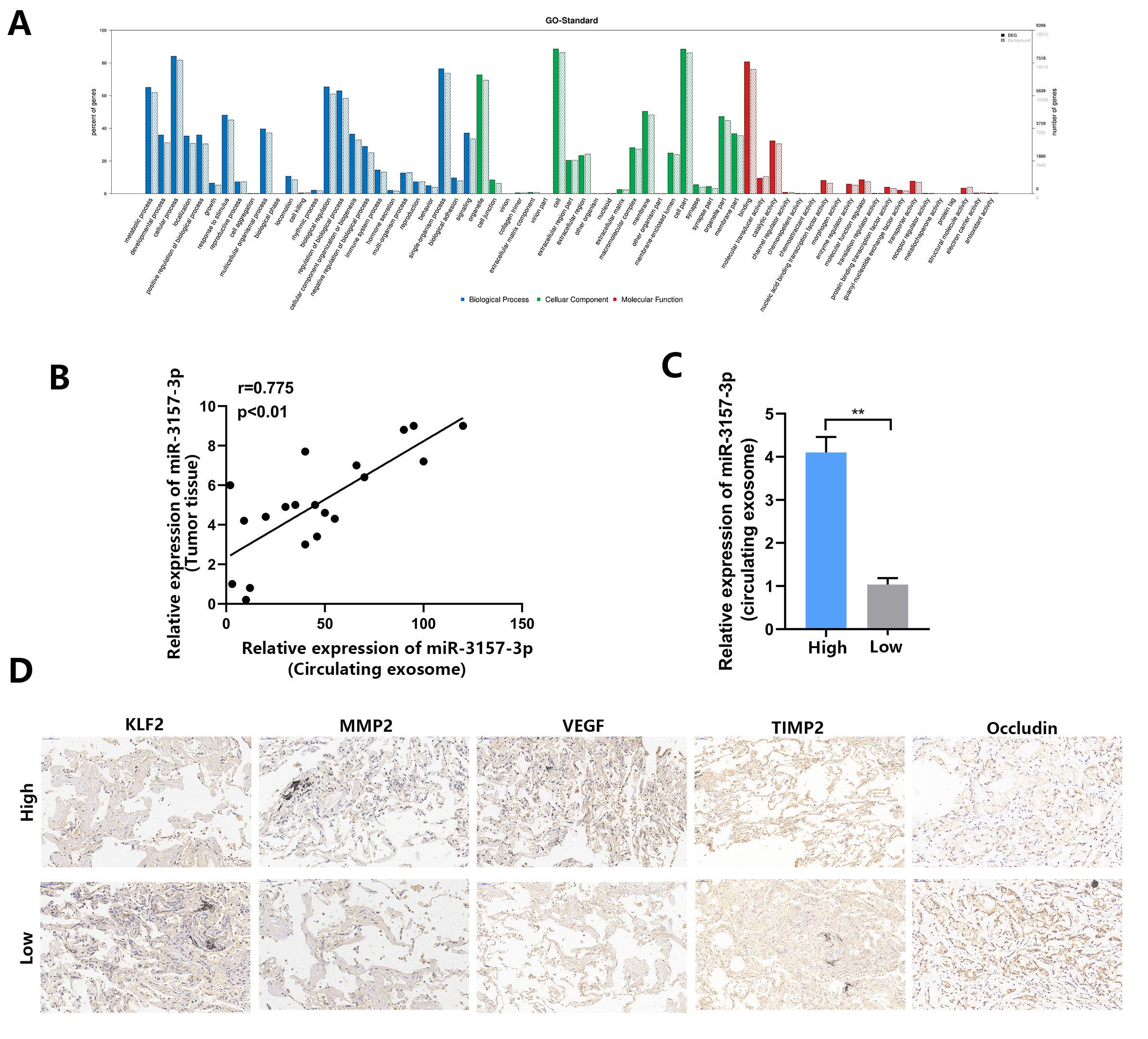

Supplement: Supplementary file 3 — Supplementary figure2 [file 41419_2021_4037_MOESM3_ESM.tif]

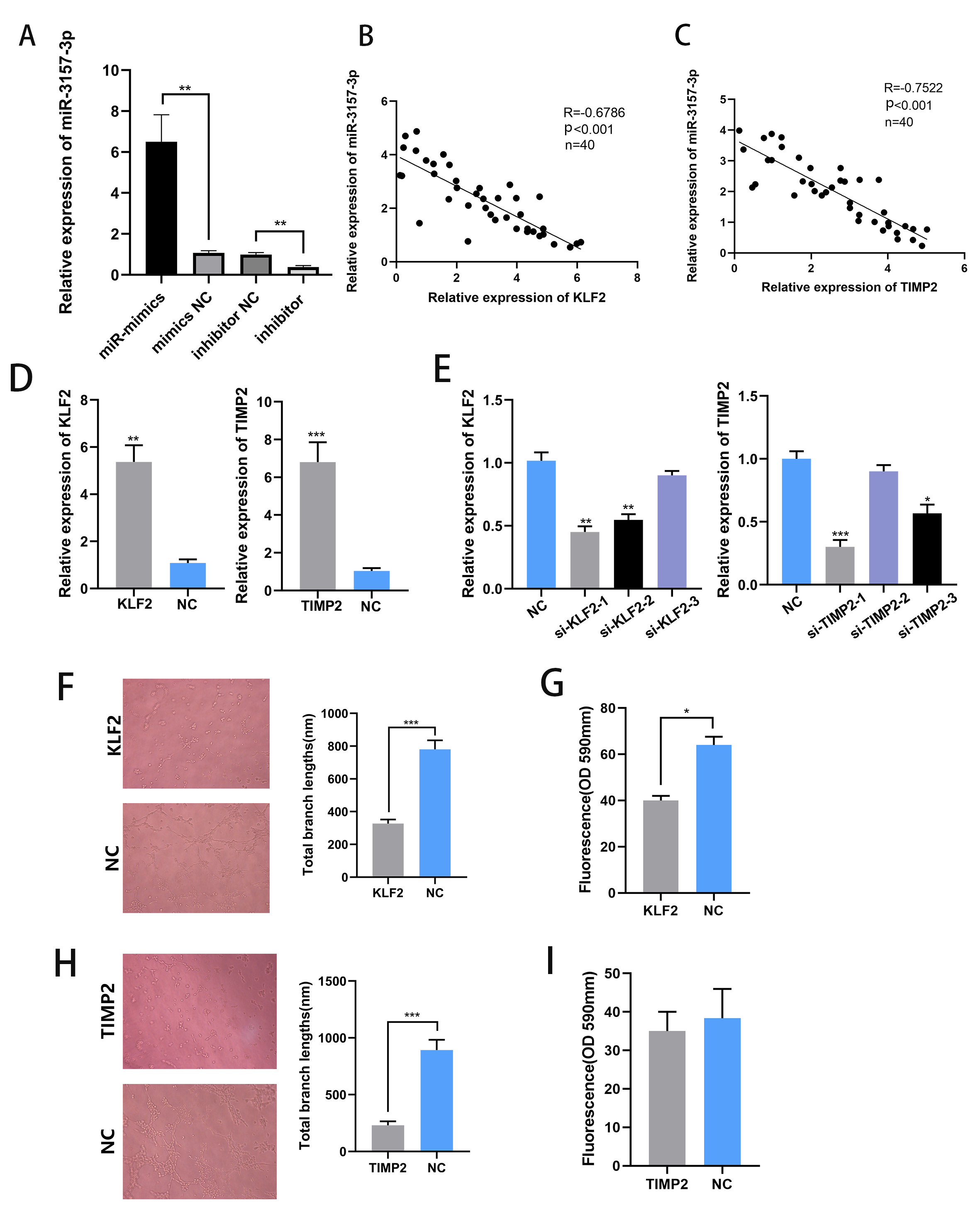

Supplement: Supplementary file 4 — Supplementary figure3 [file 41419_2021_4037_MOESM4_ESM.tif]

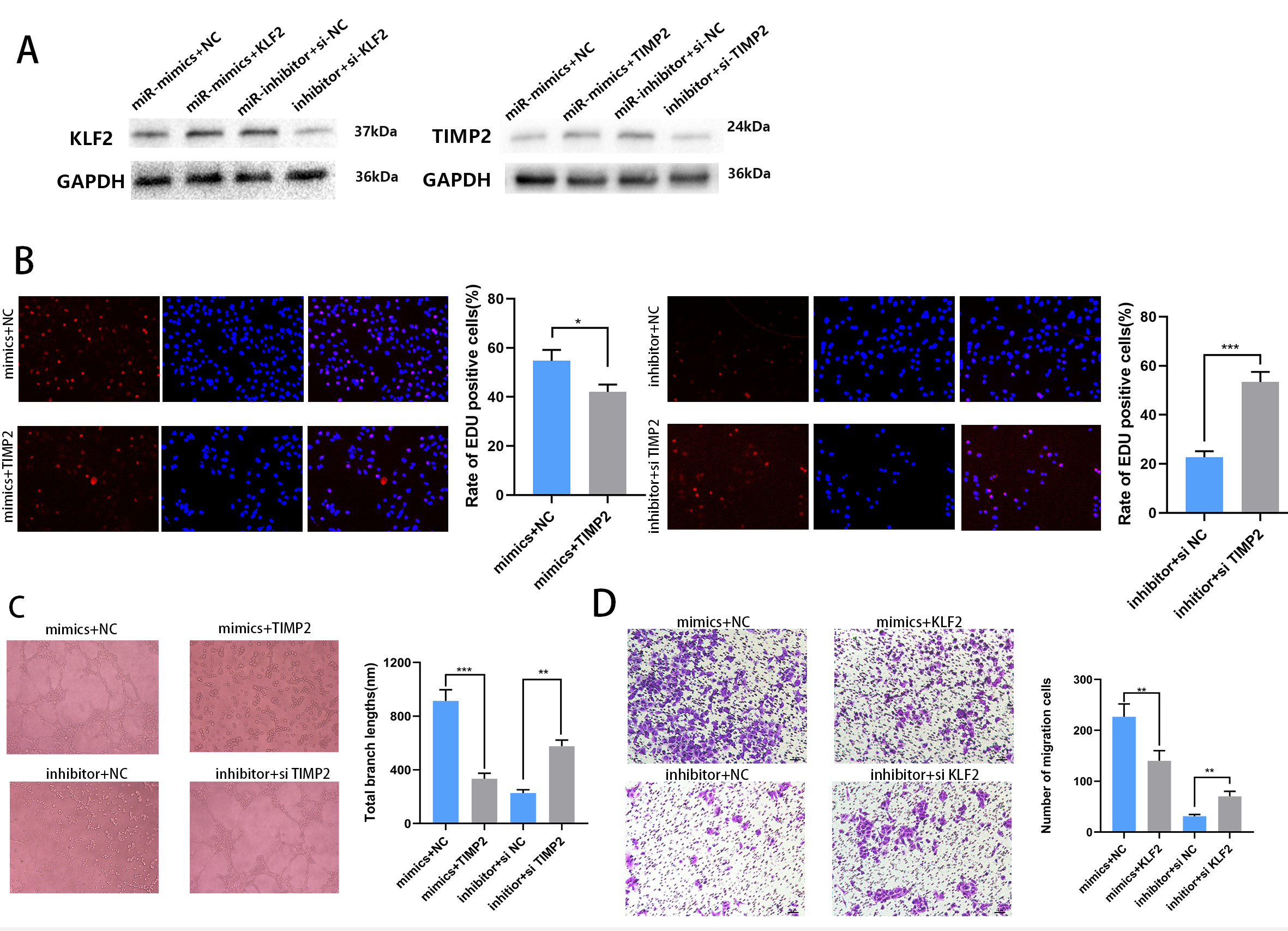

Supplement: Supplementary file 5 — Supplementary figure4 [file 41419_2021_4037_MOESM5_ESM.tif]
